# Targeted expression of heme oxygenase-1 in satellite cells improves skeletal muscle pathology in dystrophic mice

**DOI:** 10.1186/s13395-024-00346-2

**Published:** 2024-06-12

**Authors:** Urszula Florczyk-Soluch, Katarzyna Polak, Sarka Jelinkova, Iwona Bronisz-Budzyńska, Reece Sabo, Subhashini Bolisetty, Anupam Agarwal, Ewa Werner, Alicja Józkowicz, Jacek Stępniewski, Krzysztof Szade, Józef Dulak

**Affiliations:** 1 Department of Medical Biotechnology, Faculty of Biochemistry, Biophysics and Biotechnology, Jagiellonian University, Gronostajowa 7, 30-387 Kraków, Poland; 2 Department of Medicine, University of Alabama at Birmingham, Birmingham, AL USA; 3 Nephrology Research and Training Center, University of Alabama at Birmingham, Birmingham, AL USA; 4 Laboratory of Stem Cells Biology, Faculty of Biochemistry, Biophysics and Biotechnology, Jagiellonian University, Kraków, Poland

**Keywords:** Heme oxygenase-1, Duchenne muscular dystrophy, Satellite cells, Skeletal muscle regeneration

## Abstract

**Background:**

Adult muscle-resident myogenic stem cells, satellite cells (SCs), that play non-redundant role in muscle regeneration, are intrinsically impaired in Duchenne muscular dystrophy (DMD). Previously we revealed that dystrophic SCs express low level of anti-inflammatory and anti-oxidative heme oxygenase-1 (HO-1, *HMOX1*). Here we assess whether targeted induction of *HMOX1* affect SC function and alleviates hallmark symptoms of DMD**.**

**Methods:**

We generated double-transgenic mouse model (mdx;*HMOX1*^Pax7Ind^) that allows tamoxifen (TX)-inducible *HMOX1* expression in Pax7 positive cells of dystrophic muscles. Mdx;*HMOX1*^Pax7Ind^ and control mdx mice were subjected to 5-day TX injections (75 mg/kg b.w.) followed by acute exercise protocol with high-speed treadmill (12 m/min, 45 min) and downhill running to worsen skeletal muscle phenotype and reveal immediate effects of HO-1 on muscle pathology and SC function.

**Results:**

*HMOX1* induction caused a drop in SC pool in mdx;*HMOX1*^Pax7Ind^ mice (vs. mdx counterparts), while not exaggerating the effect of physical exercise. Upon physical exercise, the proliferation of SCs and activated CD34^−^ SC subpopulation, was impaired in mdx mice*,* an effect that was reversed in mdx;*HMOX1*^Pax7Ind^ mice, however, both in vehicle- and TX-treated animals. This corresponded to the pattern of HO-1 expression in skeletal muscles. At the tissue level, necrotic events of selective skeletal muscles of mdx mice and associated increase in circulating levels of muscle damage markers were blunted in HO-1 transgenic animals which showed also anti-inflammatory cytokine profile (vs. mdx).

**Conclusions:**

Targeted expression of *HMOX1* plays protective role in DMD and alleviates dystrophic muscle pathology.

**Supplementary Information:**

The online version contains supplementary material available at 10.1186/s13395-024-00346-2.

## Introduction

Duchenne muscular dystrophy (DMD) is an X-linked neuromuscular genetic disorder of poor prognosis with 1 per 5000 male births prevalence. Clinically DMD manifests as progressive skeletal muscle weakness and deterioration in motor performance leading to difficulties in walking and eventually wheelchair requirement around the age of 12 [1].

DMD originates from the mutation in dystrophin gene (*DMD*) and the lack of functional protein scaffolding submembranous components in striated muscle cells. Unmet mechanical demands result in the fragility of cortical cytoskeleton, disruption of myocyte integrity upon contraction, and myofiber degeneration triggering inflammatory reaction, one of the major hallmarks of DMD. At 3–8 weeks of age skeletal muscles of dystrophic *mdx* mice show massive necrosis followed by vigorous regeneration. Such cycles of muscle damage and regeneration repeat during this intense growth period and from ⁓12 weeks of age onwards, the pathology stabilizes with continuous degeneration (akin to DMD patients) seen solely in diaphragm, which recapitulates the most the human condition. Persistent pathological progression is observed after the age of 12 months, however, adult muscles typically present only mild necrosis while modestly increased fibrosis [1–3].

Adult muscle-resident myogenic stem cells, satellite cells (SCs), under trigger-free conditions remain quiescent in a niche on the surface of the muscle fiber, beneath the ensheathing basal lamina. In response to muscle deterioration SCs are activated to play an essential role in regeneration of ruptured fibers generating committed myogenic progenitor cells (myoblasts) [4]. In parallel, a subset of activated SCs self-renew and reinstate quiescence to replenish the stem cell reservoir [4–6]. The high intensity physical exercise that induces muscle insult is associated with SC activation and proliferation during the acute phases of injury [7–9].

Both, direct *DMD* mutation-related, and indirect, dystrophic environment-caused changes in SC biology are involved in DMD progression [10, 11]. SCs express high levels of dystrophin that mediates cell polarity, critical for asymmetric divisions [11]. Increased number of SCs in DMD may be related to the loss of dystrophin-dependent polarity resulting in inefficient generation of committed myogenic progenitors [11].

Heme oxygenase-1 (HO-1, encoded by *HMOX1*), an enzyme of anti-inflammatory and anti-oxidative potential due to the products of heme degradation [12] is involved in the response to skeletal muscle injury, being expressed mainly in leukocytes [13, 14]. Enhanced level of HO-1 is detected also in limb skeletal muscles and diaphragm of mdx mice, both in myofibers and inflammatory cells, and in myoblasts differentiated from DMD patients-derived human-induced pluripotent stem cells (hiPSCs) [15]. More recently, HO-1 has been linked with postnatal differentiation of stem and progenitor cells [14, 16, 17], but there is a gap to be filled about HO-1 involvement in SC proliferation and differentiation potential as so far condition-dependent effect of HO-1 on myogenic precursors were reported [14, 15, 17].

Our previous data showed that the genetic ablation of *Hmox1* in dystrophic mice (*Hmox1*^−/−^mdx) aggravated muscle damage, increased inflammation and was associated with impaired exercise capacity [15]. In parallel, no significant changes of SC function were detected, likely due to already reduced expression of *Hmox1* in dystrophic SCs (in contrast to muscle fibers) [15]. In this study, we sought to determine the effect of HO-1 in dystrophic SCs and its contribution to the pathogenesis of DMD. Towards this end, we generated double-transgenic mouse model (mdx;*HMOX1*^Pax7Ind^) that allows tamoxifen (TX)-inducible *HMOX1* expression in SCs of dystrophic muscles. The high intensity physical exercise regimen was applied to reveal immediate effects of HO-1 on muscle pathology and SC function.

## Methods

### Mice

ROSA26LSL-HO1 (B6-*Gt(ROSA)26Sor*^*tm(HMOX1)*^) mice with human HO-1 cDNA cloned downstream of a floxed stop codon in the endogenous ROSA26 locus were a kind gift from prof. Anupam Agarwal [18]. Pax7-CreER^T2^ (B6.Cg-Pax7tm1(cre/ERT2)Gaka/J) mice with an internal ribosome entry site (IRES)-CreER^T2^ fusion protein with Cre recombinase fused to a triple mutant form of the human estrogen receptor, inserted downstream of the stop codon of *Pax7* gene were purchased form the Jackson laboratory. Cre-ER^T2^ fusion gene activity is inducible by TX administration.

**Fig. 1:**
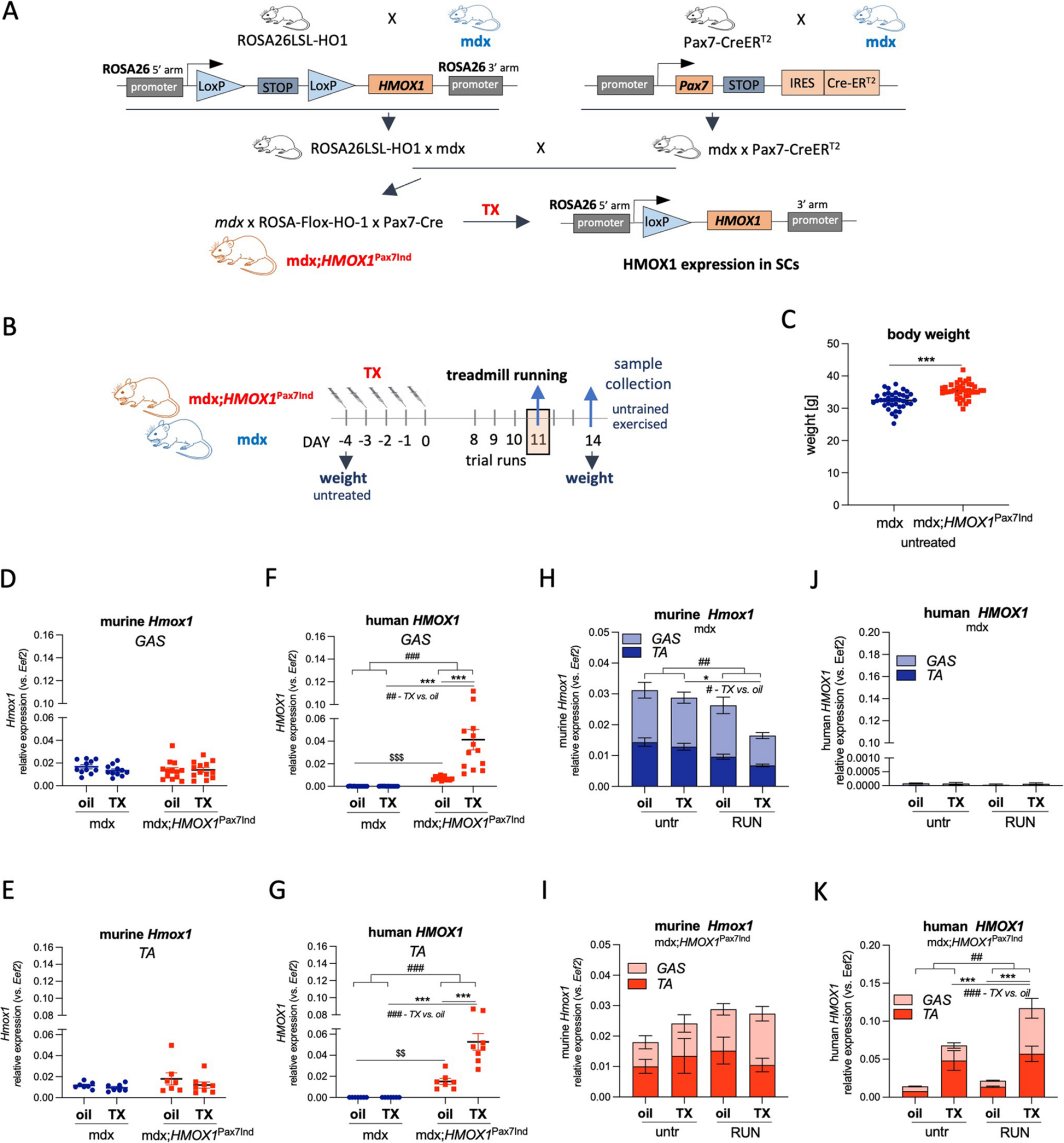
*HMOX-1* expression in induced by TX in skeletal muscles of mdx;*HMOX1*^Pax7Ind^ mice. **A** A scheme of generation of mdx;*HMOX1*^Pax7Ind^ mice with tamoxifen (TX) inducible *HMOX1* expression in SCs. **B** An experimental scheme of TX injections to mdx;*HMOX1*^Pax7Ind^ and mdx mice. Body weight of (**C**) untreated animals (*n* = 38). Relative expression of (**D**, **E**) murine *Hmox1* and (**F**, **G**) human *HMOX1* in *caput gastrocnemius* (*GAS*) (*n* = 11–13) and *tibialis anterior (TA)* (*n* = 7–8) upon TX administration (untreated + RUN), qPCR*.* (H–K) The effect of physical exercise on HO-1 level at day 3 after exercise. Relative expression of *Hmox1* (**H**, **I**)*/ HMOX1* (J,K) in *GAS* (*n* = 5–8) and *TA* (*n* = 3–4) in (**H**, **J**) mdx and (**I**, **K**) mdx;*HMOX1*^Pax7Ind^ mice, qPCR. Data is presented as mean^±^ SEM. ##* p* < 0.01, ###* p* < 0.001—*Two-way* ANOVA variation; * *p* < 0.05, ** *p* <  < 0.01, *** p < 0.001 by *Two-way* ANOVA with Tukey’s post hoc test; $$ *p* < 0.01, $$$—*p* < 0.001 by unpaired two-tailed Student’s *t* test

A female mdx (C57BL/10ScSn-Dmdmdx/J) was crossed with ROSA26LSL-HO1 (male) (crossing 1) and, in parallel, Pax7-CreER^T2^ (male) (crossing 2). A female from crossing 1 and male from crossing 2 were bred together to obtain double-transgenic mice with TX-inducible *HMOX1* expression in SCs – mdx;*HMOX1*^Pax7Ind^ (B6.Cg-Dmdmdx*Gt(ROSA)26Sor*^*tm(Hmox−1)*^Pax7^tm1(cre/ERT2)^) (Fig. 1A). 13–16-week-old males were used for experiments.

Animal experiments were performed after approval (73/2019, 26/2020, 315/2020) by the 2nd Institutional Animal Care and Use Committee (IACUC) in Krakow, Poland, according to Polish and European legislation and according to the ARRIVE guidelines. Mice were housed under specific pathogen free (SPF) conditions in individually ventilated cages with a 14 h/10 h light/dark cycle and were kept on a normal, chow diet with water and food available ad libitum.

### Tamoxifen administration and treadmill running

Mice were given intraperitoneal injection of TX (Sigma, 75 mg/kg b.w. in ~ 100 µl of corn oil) once a day for 5 consecutive days or with corn oil (~ 100 µl) as a control. TX stock (20 mg/ml) was incubated in 37 ◦C with shaking until completely dissolved before use.

Upon 7-day waiting period mice were subjected to physical exercise on a treadmill (Exer 3/6, Columbus Instruments). After familiarization trainings (8 m/min, 15 min) on three consecutive days, the next day mice were run according to the following program: 0—6 m/min—2 min, 6—8 m/min—2 min, 8—10 m/min—2 min, 10—12 m/min—45 min with downhill inclination of 15° (acute exercise protocol). At day 3 upon exercise regimen (2 weeks after the last TX injection) mice were euthanized with carbon dioxide and the blood was collected from the heart of unconscious animals. Skeletal muscles from one limb were collected for FACS analysis. *Caput gastrocnemius* and *tibialis anterior* from the second limb as well as diaphragmae were dissected for histological procedures and gene expression analysis.

### Serum LDH and CK activity

The heart blood collected to 1.5 ml Eppendorf tube was left for 10–30 min at room temperature (rt) for clotting. Upon centrifugation (1000 × *g*, 10 min, 4 ◦C) serum was collected and stored at -80 ◦C.

Serum samples were diluted 10 × in water. The activity of lactate dehydrogenase (LDH) and creatine kinase (CK) was measured using the diagnostic Liquick Cor-LDH and Liquick Cor-CK kit (Cormay), respectively, according to manufacturer’s protocol.

### RT-qPCR

Skeletal muscles: *caput gastrocnemius, tibialis anterior* and *diaphragmae* were collected into RNA*later* tissue storage reagent (Sigma-Aldrich), frozen in liquid nitrogen and stored at –80 ◦C. Tissue samples were homogenized in phenol/guanidine-based QIAzol Lysis Reagent (Qiagen). Total RNA was isolated by organic extraction upon addition of chloroform followed by isopropanol precipitation. Ethanol-washed RNA pellets were redissolved in RNase-free water. RNA concentration was determined by NanoDrop Spectrophotometer (ThermoFisher Scientific).

Reverse transcription (RT) of mRNA was performed using 500 ng of total RNA and RevertAid reverse transcriptase (ThermoFisher Scientific) according to vendor’s instructions and carried out in ProFlex PCR System (Applied Biosystems). cDNA (40 ng per sample) was used for quantitative PCR (qPCR) with Sybr® Green JumpStart™ Taq ReadyMix™ (Sigma-Aldrich) and specific primers (Table 1). qPCR was carried out in StepOne Plus Real-Time PCR System (Applied Biosystems) and analyzed with StepOne Software v2.3. *Eef2* was used as a housekeeping gene for relative quantification of gene expression based on 2^–ΔCt^ method.

**Table 1:** The sequences of primers used for the determination of gene expression at mRNA level by qPCR

*Eef2/EEF2*	F—5’- TCAGCACACTGGCATAGAGGC—3’
	R—5’- GACATCACCAAGGGTGTGCAG—3’
*HMOX1*	F—5’- CATGACACCAAGGACCAGAG—3’
	R—5’- AGTGTAAGGACCCATCGGAG—3’
*Hmox1*	F—5’- GGTGATGGCTTCCTTGTACC—3’
	R—5’- AGTGAGGCCCATACCAGAAG—3’
*Il10*	F—5’- GGTTGCCAAGCCTTATCGGA—3’
	R—5’- ACCTGCTCCACTGCCTTGCT—3’

### Skeletal muscle isolation for flow cytometry

Skeletal muscles from one limb (*caput gastrocnemius, soleus, tibialis anterior, EBL, quadriceps, adductor*) were collected, cut with scissors and a scalpel, and digested with 7 ml mixture of collagenase IV (5 mg/ml) and dispase (1.2 U/ml) in PBS (30 min at 37 ◦C with shaking). Muscle samples were then washed with 10% FBS in PBS and centrifuged (600 × *g*, 10 min, 4 °C). Tissue pellets were resuspended in 2% FBS in PBS, filtered through 100 µm pores, centrifuged (600 × *g*, 10 min, 4 ◦C) and washed again in 2% FBS in PBS. Finally, pellets were resuspended in 150 µl of 2% FBS in PBS without ions and used for FACS analysis.

### FACS analysis

For the analysis of muscle SCs, 100 µl of skeletal muscle samples were incubated (30 min, 4 ◦C) with a mixture of anti-mouse antibodies against: CD31 (1:30, APC, BD Pharmingen, cat: 551,262), CD45 (1:30, APC-Alexa Fluor 780, BD Pharmingen, cat: 47–0451-82), CD34 (1:30, Alexa Fluor 700, eBioscience, cat: 56–0341-82), Ly6 A/E (Sca-1) (1:30, PE-Cy7, D7, eBioscience, ref: 25–5981-82) and α7 integrin (1:15, PE, R&D Systems, FAB 3518 A ref: FAB3518P). Upon washing in 2% FBS containing PBS samples were centrifuged (600 × *g*, 10 min, 4 °C) and fixed and permeabilized using BD Intrasure™ kit (BD Biosciences) according to vendor’s instruction for staining intracellular markers. Rabbit anti-HO-1 (1:100, Enzo, cat: ADI-SPA-894-F) and anti-Ki67 (1:30, Alexa Fluor 488, ThermoFisher Scientific, cat: 53–5698-82) antibodies were then applied (30 min, rt) followed by incubation with secondary anti-rabbit IgG AlexaFluor 568 (1:200, ThermoFisher Scientific) (30 min, rt). Upon washing in 2% FBS containing PBS followed by centrifugation (600 × *g*, 10 min, 4 °C) samples were additionally stained with 100 µL Hoechst33342 (1 μg/ml, Sigma-Aldrich) for 10 min (rt).

The stained cells were analyzed using LSRFortessa flow cytometer with FACSDiva v8.1 software (BD Biosciences). To investigate mononucleated cell populations doublets exclusion was done based on elimination of events with increased FSC-Width and SSC-Width values followed by Hoechst-positive gating.

### Histological procedures

Dissected skeletal muscle tissues (*caput gastrocnemius, tibialis anterior, diaphragmae*) were mounted in OCT Compound (Leica), frozen in liquid nitrogen–cooled isopentane and stored at –80 ◦C until cryostat sectioning. Frozen Sects. (8 μm thick, kept at -20 ◦C) were used for hematoxylin and eosin (H&E, Sigma-Aldrich) staining to visualize tissue morphology, inflammatory infiltration, and necrosis, and for immunofluorescence (PAX7, HO-1, IgG/IgM).

Immunofluorescent staining of Pax7/HO-1 was performed on *caput gastrocnemius* frozen sections. Fixation was performed with 4% paraformaldehyde (25 min, rt) and with methanol (5 min, -20 °C). Citrate buffer (10 mM citric acid in water, pH6) was used for antigen retrieval following 30 min cooling down from boiling buffer. Sections were then blocked with 2.5% BSA containing M.O.M.® (Mouse on Mouse) blocking reagent (Vector Laboratories) for 30 min at rt. Primary antibodies: murine anti-PAX7 (1:100, DSHB) and rabbit anti-HO-1 (1:300, Enzo) were diluted in 0.1% BSA in PBS and added to the sections for overnight incubation at 4 °C in high humidity chamber. Secondary antibodies: anti-mouse IgG Alexa Flour 488 and anti-goat IgG Alexa Fluor 594 (1:500 each, ThermoFisher Scientific) were diluted in 0.1% BSA in PBS and added for 90 min at rt. Finally, the sections were counterstained with DAPI for 10 min, mounted with fluorescent mounting medium (Dako) and covered with a cover glass. Pictures were taken with LSM-880 meta laser scanning confocal microscope (Zeiss).

For immunofluorescent staining of IgG/IgM frozen sections were air-dried for 15–30 min (rt) and blocked with 10% goat serum for 1 h. Rabbit anti-laminin primary antibody (1:500, Sigma-Aldrich) diluted in 1% goat serum in PBS was applied for 1 h at 37 °C, following incubation with a mixture of anti-rabbit IgG Alexa Fluor 568 (1:500, ThermoFisher Scientific) and goat anti-mouse IgG/IgM (H + L) Alexa Fluor 488 (1:400, ThermoFisher Scientific) with Hoechst33342 (1 μg/ml, Sigma-Aldrich) for 30 min at 37 °C. Sections were mounted with fluorescent mounting medium (Dako) and covered with a cover glass. Scans of the whole tissue sections and representative pictures were done with Leica DMi8 microscope with CMOS Leica MC170 HD camera.

### Statistics

Numerical data is presented as mean ± standard error (SEM). The outliers were excluded based on the Grubb’s test. The *n* number refers to the number of mice used for experiment and is indicated in the figures legends. Two-way ANOVA (for multiple group comparisons), followed by Tukey’s post hoc test was used to determine the statistical significance (*p* < 0.05). Student’s *t-test* was used additionally for direct comparison of two groups that were not significantly different using ANOVA tests. GraphPad 8™ software was used for all statistical analysis. *p* values 0.05 – 0.1 were considered as a statistical tendency.

## Results

### Generation of transgenic dystrophic mice with SC-specific *HMOX1* expression

To obtain inducible HO-1 expression in SCs of dystrophic mice, we generated double-transgenic mice on mdx background (Fig. 1A). In brief, ROSA26LSL-HO1 mice with human *HMOX1* cDNA downstream of a floxed stop codon cloned into the endogenous ROSA26 locus were generated previously [18]. To excise the stop codon and enable *HMOX1* transgene expression, we introduced Cre recombinase in Pax7-CreER^T2^ mice with an internal ribosome entry site (IRES)-CreER^T2^ fusion construct inserted downstream of the stop codon of *Pax7* gene. Pax7, a SC marker, is expressed and functional, while Cre-ER^T2^ activity is inducible by tamoxifen (TX), a selective estrogen receptor regulator, that allows Cre nuclear localization. Dystrophic background was introduced by mdx mice breeding with Rosa26LSL-HO1 and Pax7-CreER^T2^ mice (Fig. 1A).

Obtained double-transgenic mouse model (mdx;*HMOX1*^Pax7Ind^) allows TX-inducible, Cre-mediated recombination at the LoxP sites, and *HMOX1* expression in Pax7 positive cells. In parallel, TX-treated mdx mice served as control of potential side effects of TX.

The body weight of untreated animals (before experiment) was higher in mdx;*HMOX1*^Pax7Ind^ than in mdx mice (Fig. 1C). However, in both genotypes neither TX- nor exercise-dependent effect on body weight was detected as measured before and two weeks after the last TX injection (Suppl. Figure 1A). In addition the total weight of hind limb skeletal muscles (*caput gastrocnemius, soleus, tibialis anterior, EBL, quadriceps, adductor*) collected from one limb measured at the end of experiment was comparable between groups of both genotypes (Suppl. Figure 1B).

### TX induces *HMOX1* expression in skeletal muscles of transgenic mdx;*HMOX1*^Pax7Ind^ mice

Transgenic and control mice aged 13–16 weeks were subjected to the acute physical exercise protocol with high-speed treadmill (12 m/min, 45 min) and downhill running [19] to worsen skeletal muscle phenotype and reveal immediate effects of HO-1 on muscle pathology and SC activation. Mdx;*HMOX1*^Pax7Ind^ and mdx mice were given 5-day TX injections and upon 7-day waiting subjected to exercise protocol (Fig. 1B).

Comparable mRNA level of murine *Hmox1* was detected in mdx mice and transgenic mdx;*HMOX1*^Pax7Ind^ mice both in *caput gastrocnemius* (*GAS*) (Fig. 1D) and *tibialis anterior* (*TA*) (Fig. 1E). TX administration induced the expression of human *HMOX1* (over oil group) specifically in skeletal muscles of transgenic mice, *GAS* (Fig. 1F) and *TA* (Fig. 1G). Nonetheless, *HMOX1* expression in oil group of both transgenic muscles was higher than in oil-treated mdx counterparts suggesting either the leakage of the Cre-LoxP system and TX-independent action of Cre, or *HMOX1* DNA detection (Fig. 1F,G).

In mdx mice, murine *Hmox1* was diminished in skeletal muscles on day 3 upon treadmill exercise, with TX exacerbating this decline (Fig. 1H) and opposite trends seen in mdx;*HMOX1*^Pax7Ind^ mice (Fig. 1I). One may consider the regulation of *Hmox1* by human HO-1 activity in transgenic mice or strain-dependent pattern of *Hmox1* regulation.

No *HMOX1* expression was detected in mdx mice (Fig. 1J). The induction of human *HMOX1* expression after TX treatment was detected in skeletal muscles of untreated transgenic mice and further increased upon exercise (Fig. 1K). This might be related to enhanced activity of ROSA promoter after exercise but requires more in-depth analysis.

### TX increases HO-1 level in SCs of transgenic mdx;*HMOX1*^Pax7Ind^ mice

**Fig. 2:**
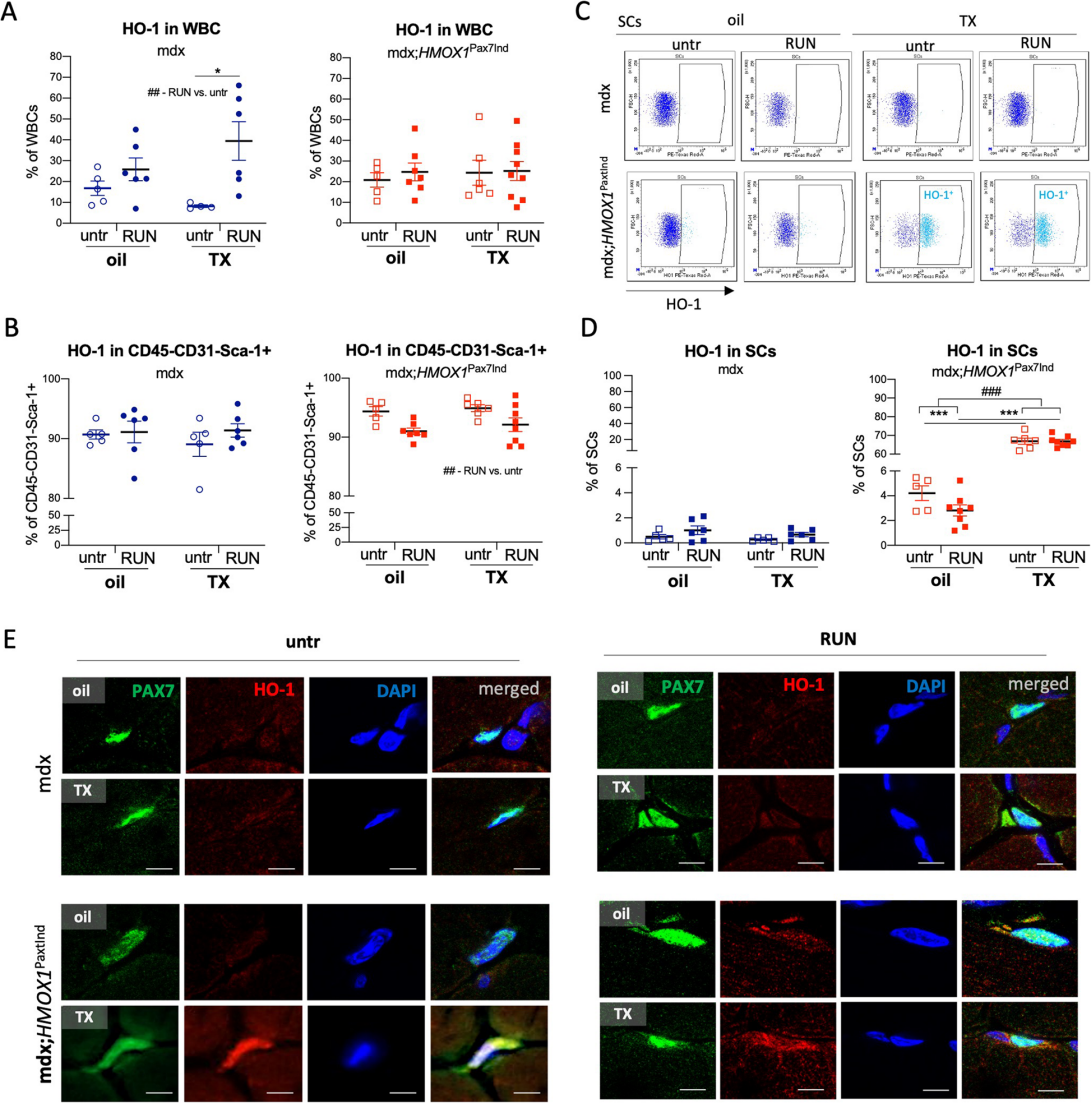
*HMOX1* expression in induced by TX in SCs of mdx;*HMOX1*^Pax7Ind^ mice. Quantitative analysis of the percentage of HO-1 positive cells among (**A**) CD45 + CD31- (WBCs), (**B**) CD45-CD31-Sca-1 + cells, and (**C**) representative plots and (**D**) quantitative analysis of the percentage of HO-1 positive cells among SCs in mdx and mdx;*HMOX1*^Pax7Ind^ hind limb skeletal muscles. Flow cytometry (*n* = 5–8). Data is presented as mean^±^ SEM. ##* p* < 0.01, ###* p* < 0.001—Two-way ANOVA interaction; *** p < 0.001 by Two-way ANOVA with Tukey’s post hoc test. (**F**) HO-1 is expressed and colocalizes with Pax7 expression in TX-treated mdx;*HMOX1*^Pax7Ind^ but not in mdx mice. Immunofluorescence of Pax7 (green), HO-1 (red) and nuclei (DAPI, blue). Scale bar: 5 μm

HO-1 protein was detected in ~ 20% of all nucleated cells (Suppl. Figure 2A,B) and at similar proportion in white blood cells (WBCs, CD45^+^CD31^−^) (Fig. 2A) in hind limb skeletal muscles (*GAS, TA, soleus, EBL, quadriceps, adductor*) of transgenic as well as control mdx mice. The number of HO-1^+^ cells in WBC population increased further by physical exercise in mdx mice (Fig. 2A). Interestingly, the majority (~ 90%) of CD45^−^CD31^−^Sca-1^+^, that may comprise side population SCs [20] or myoblasts [21], of both strains expressed HO-1 (Fig. 2B, Suppl. Figure 2A). However, in the transgenic animals we detected exercise-specific drop of CD45^−^CD31^−^Sca-1^+^ population expressing HO-1, not seen in mdx counterparts (Fig. 2B).

The specific induction of HO-1 was detected in SCs (CD45^−^CD31^−^Sca-1^−^α7integrin^+^) (Suppl. Figure 2A), upon TX administration both in untrained and exercised transgenic mice (vs. oil treatment and mdx counterparts) (Fig. 2C,D). As in the case of *HMOX1* mRNA level in *GAS* and *TA* (Fig. 1F,G), the HO-1 protein in SCs could be already detected in oil-treated mdx;*HMOX1*^Pax7Ind^ mice (vs. mdx) (Fig. 2D). Finally, HO-1 protein was detected in Pax7-positive cells in TX-treated mdx;*HMOX1*^Pax7Ind^ but not in mdx mice (Fig. 2E) verifying SC-targeted HO-1 induction in mdx;*HMOX1*^Pax7Ind^ skeletal muscles.

### HO-1 induction decreases SC pool in mdx;*HMOX1*^Pax7Ind^ mice

**Fig. 3:**
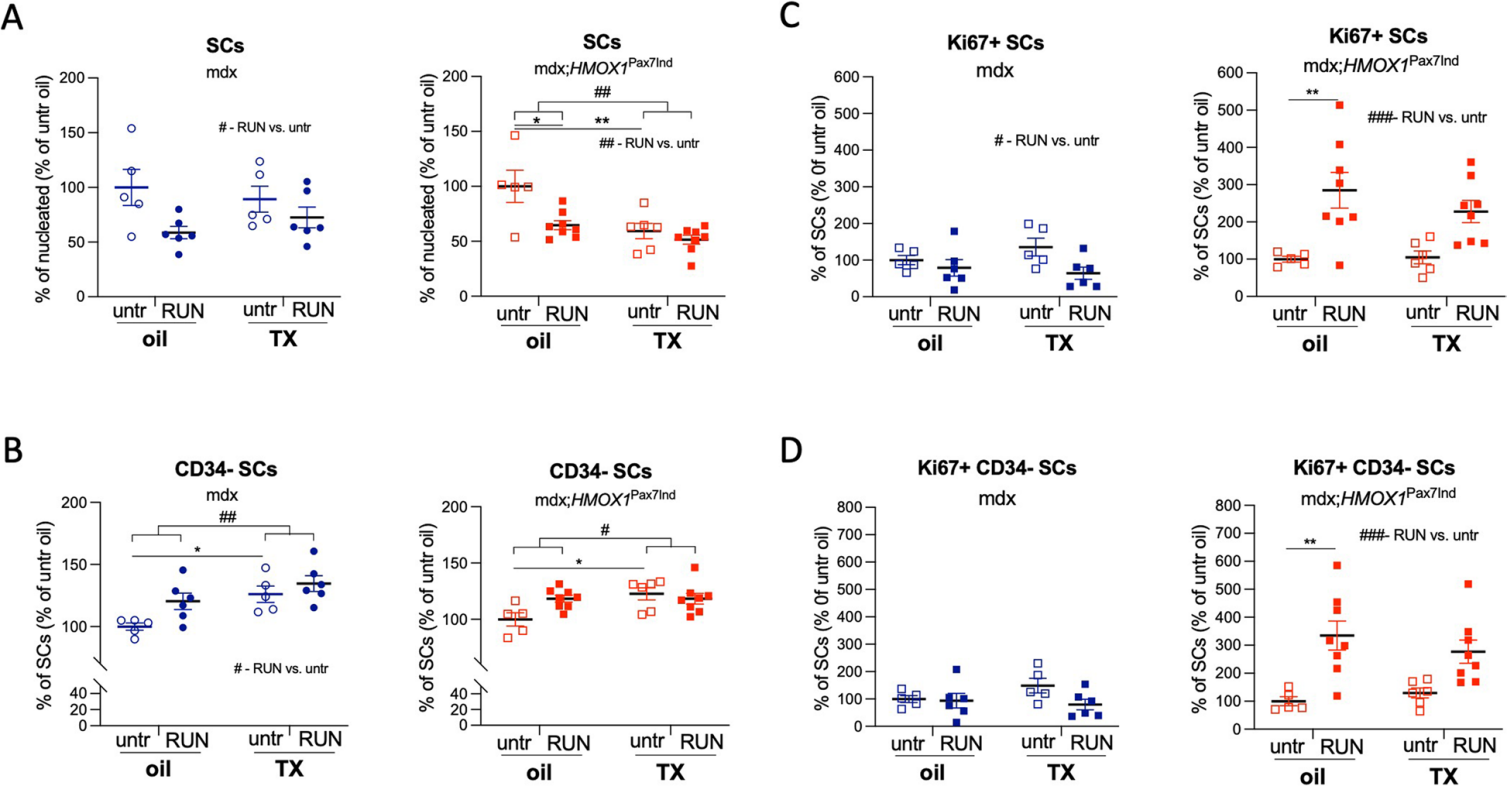
The effect of HO-1 induction on SC pool, activation and proliferation. **A** HO-1 decreases SC pool in hind limb muscles of mdx;*HMOX1*^Pax7Ind^ mice. Quantitative analysis of the percentage of SCs in muscles of mdx;*HMOX1*^Pax7Ind^ and mdx mice. Flow cytometry (*n* = 5–8). **B** SC activation (based on the loss of CD34) and proliferation (Ki67 +) of (**C**) SCs and (**D**) CD34- SC subpopulation in hind limb skeletal muscles of mdx and mdx;*HMOX1*^Pax7Ind^ mice. Flow cytometry (*n* = 5–8). Data is presented as mean^±^ SEM. #* p* < 0.05, ##* p* < 0.01, ###* p* < 0.001—Two-way ANOVA variation; * p < 0.05, ** p < 0.01 by Two-way ANOVA with Tukey’s post hoc test

The high-speed treadmill exercise regimen decreased SC pool in control, oil-treated mice of both HO-1 transgenic and control mice (Fig. 3A). HO-1 induction caused a drop in SC pool, decreasing the percentage of SCs in mdx;*HMOX1*^Pax7Ind^ mice (Fig. 3A) to the comparable extent as the physical training (Fig. 3A).

Studies have shown that high intensity physical exercise that induce muscle insult is associated with SC activation and increase in numbers during the acute phases of injury [7–9]. In HO-1 transgenic (mdx;*HMOX1*^Pax7Ind^) and control mdx mice, SC activation associated with the loss of CD34, a marker of quiescent SCs [22], increased in response to physical exercise in a comparable manner (Fig. 3B). CD34^−^ SCs were also more frequent in both genotypes upon TX administration suggesting HO-1-independent stimulation of SC activity (Fig. 3B).

No HO-1 specific effect was detected in populations defined upstream SC gating: CD45^−^CD31^−^ (Suppl. Figure 3A), CD45^+^CD31^−^ (WBCs, Suppl. Figure 3B), CD45^−^CD31^+^ (endothelial cells, ECs, Suppl. Figure 3C), CD45^−^CD31^−^Sca-1^−^ (Suppl. Figure 3D) or CD45^−^CD31^−^Sca-1^+^. However, TX treatment caused a decrease of the percentage of CD45^−^CD31^−^Sca-1^+^ in mdx mice, the effect reversed by physical exercise (Suppl. Figure 3E).

Upon physical exercise, the percentage of proliferating SCs (Fig. 3C) and CD34^−^ SC subpopulation (Fig. 3D) decreased in control mdx mice*,* an effect that was reversed in mdx;*HMOX1*^Pax7Ind^ mice. Similar pattern was detected in oil-treated and TX-treated mdx;*HMOX1*^Pax7Ind^ mice that could be related to the leakage of the Cre-LoxP system (Fig. 3C,D, compare with Figs. 1F,G, 2D).

In skeletal muscles of control mdx mice, we observed exercise-dependent decrease in murine *Hmox1* expression that correlated with a drop in SC proliferation (Figs. 1H, 3C,D). In contrast, exercise induced HO-1 mRNA level in transgenic mice and also increased SC proliferation (Figs. 1I, K, 3C, D). On the other hand, no respective changes in HO-1 level was visible in SCs (Fig. 2D).

### SC-targeted *HMOX1* expression modulates skeletal muscle inflammation in dystrophic mice

**Fig. 4:**
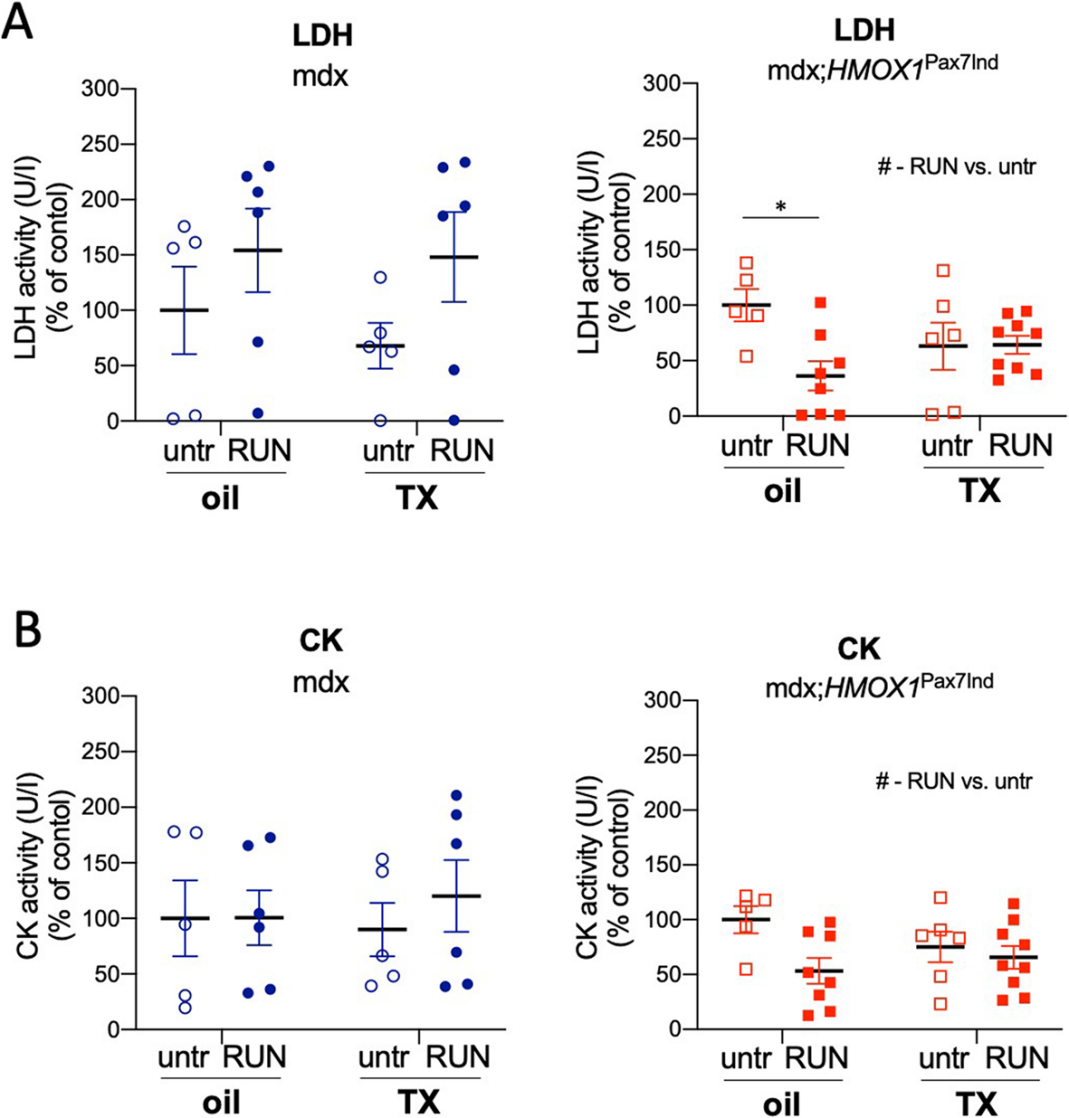
mdx;*HMOX1*^Pax7Ind^ mice show lower serum level of muscle damage markers upon physical exercise. **A** LDH and (**B**) CK activity in serum of mdx and mdx;^Pax7Ind^ mice. Colorimetric evaluation (*n* = 5–9). Data is presented as mean^±^ SEM. #* p* < 0.05—Two-way ANOVA variation; * p < 0.05 by Two-way ANOVA with Tukey’s post hoc test

Muscle-derived circulating CK and LDH serve as DMD biomarkers reflecting membrane damage, necrosis and extracellular leakage [23, 24]. Mdx;*HMOX1*^Pax7Ind^ mice showed lower serum level of muscle damage markers upon physical exercise in comparison to mdx mice (Fig. 4A,B).

**Fig. 5:**
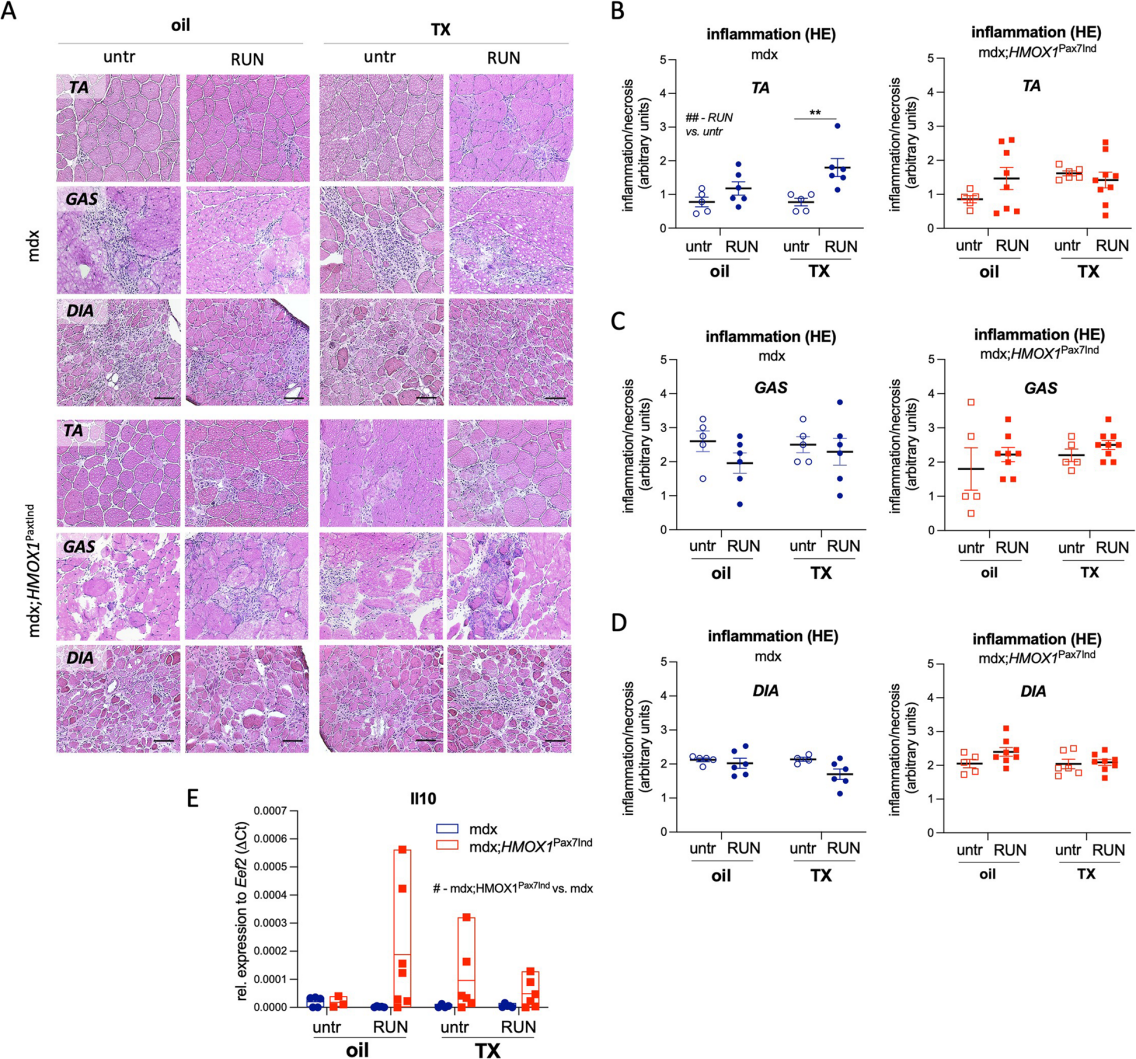
*HMOX1* induction in SCs modulates skeletal muscle inflammation in dystrophic mice. **A-E** Inflammation assessment in *tibialis anterior (TA), diaphragmae (DIA)* and *gastrocnemius (GAS)* tissue sections. (**A**) Representative images of leukocyte infiltration/necrosis in skeletal muscles. Scale bar: 100 μm. **B-D** Semiquantitative analysis in (**B**) *TA*, (**C**) *GAS* and (**D**) *DIA* (*n* = 5–9). H&E staining. Relative expression of *Il10* in *TA*. qPCR (*n* = 3–7). Data is presented as mean^±^ SEM. #* p* < 0.05, ##* p* < 0.05—*Two-way* ANOVA variation; ** p < 0.01 by Two-way ANOVA with Tukey’s post hoc test

To further outline the effect of HO-1 in SCs we characterized a dystrophic phenotype and the extent of skeletal muscle damage in mdx;*HMOX1*^Pax7Ind^ mice (vs. mdx counterparts). The percentage of WBCs (Suppl. Figure 3B) as well as ECs (Suppl. Figure 3C) in hind limb muscles assessed by flow cytometry was not changed by SC-specific HO-1 induction neither in untrained nor in exercise groups and was comparable to mdx counterparts (Suppl. Figure 3B,C). The histological analysis of *TA*, *GAS* and *DIA* muscles of mdx and mdx;*HMOX1*^Pax7Ind^ mice did not reveal significant effects of HO-1 on inflammatory response (Fig. 5A-D). However, there was an increase in inflammatory cell infiltration after exercise in *TA* of mdx mice, but not in HO-1 transgenic mice (Fig. 5B). Also, the basal inflammation extent was moderately higher in *GAS* of mdx mice than in HO-1 transgenic mice (Fig. 5A,C). In addition, HO-1 transgenic mice revealed anti-inflammatory cytokine profile. The level of antinflammatory interleukin 10 (IL-10) known to limit T cell activation and production of proinflammatory cytokines [25] increased in mdx;*HMOX1*^Pax7Ind^ mice (vs. mdx counterparts) both in response to exercise and TX administration (Fig. 5E). In parallel, TX increased IL-10 level in untrained mdx;*HMOX1*^Pax7Ind^ mice, while decreased run-induced IL-10 expression suggesting more complex inflammatory effect (Fig. 5E).

### SC-specific *HMOX1* induction attenuates skeletal muscle necrotic events upon physical exercise

**Fig. 6:**
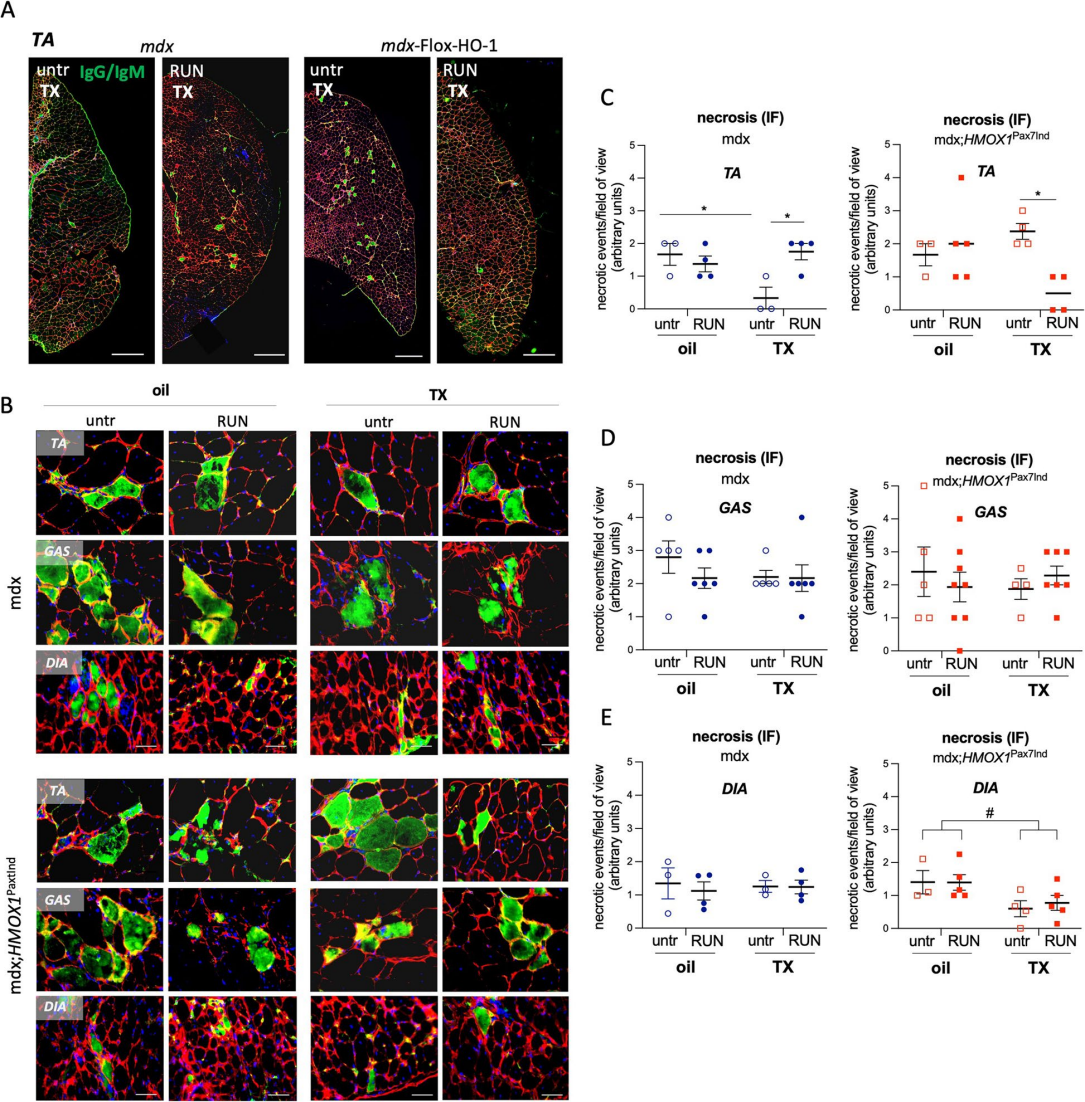
*HMOX1* induction in SCs attenuates skeletal muscle necrotic events upon physical exercise. **A-E** Necrosis assessment in *tibialis anterior (TA), gastrocnemius (GAS)* and *diaphragmae (DIA)* tissue sections of mdx and mdx;*HMOX1*^Pax7Ind^ mice*.* (**A**) Representative scans of *TA*. Scale bar: 500 μm. **B** Representative images of necrotic events in *TA, DIA and GAS*. Scale bar: 100 μm. Semiquantitative analysis in (**C**) *TA*, (**D**) *GAS* and (**E**) *DIA*. Immunofluorescent staining of IgG and IgM (green) (*n* = 3–8). Data is presented as mean^±^ SEM. #* p* < 0.05—*Two-way* ANOVA variation; * p < 0.05 by Two-way ANOVA with Tukey’s post hoc test

The analysis of skeletal muscles revealed HO-1 dependent anti-necrotic action upon physical exercise in *tibialis* of mdx;*HMOX1*^Pax7Ind^ and opposite pattern seen in TX-treated mdx mice, as evidenced by assessment of IgM and IgG accumulation (Fig. 6A-E). In untreated mdx mice necrotic events decreased in *TA* after TX treatment while rose back to control values upon physical exercise, in contrast to HO-1 transgenic mice, in which the IgG/IgM accumulation decreased significantly after exercise (Fig. 6A-C). The reverse effect of TX in mdx;*HMOX1*^Pax7Ind^ and mdx mice in *TA* (Fig. 6A-C) resembled the pattern of inflammation extent observed in *TA* stained with H&E (Fig. 5A,B). No changes were detected between experimental groups in *GAS* muscle, likely due to already high basal inflammation extent in this muscle in both strains (Fig. 6B,D). HO-1-dependent anti-necrotic action was detected also in diaphragm of mdx;*HMOX1*^Pax7Ind^ both in untrained and exercised transgenic mice in comparison to mdx counterparts (Fig. 6B,E).

## Discussion

This study points at the role of HO-1 in dystrophic SCs and beneficial effect against skeletal muscle pathology in dystrophic mice.

In the hind limb skeletal muscles of mdx;*HMOX1*^Pax7Ind^ mice, the induction of SC-specific expression of *HMOX1* decreased SC pool suggesting its role in stimulation of muscle differentiation, without affecting SC activation, that was increased by TX in HO-1 independent manner. In mdx;*HMOX1*^Pax7Ind^ mice we detected physical exercise-induced SC proliferation and opposite pattern in control mdx mice. As similar effect was detected in vehicle and TX-treated transgenic mice, direct involvement of HO-1 is uncertain. The changes in SC proliferation corresponded to the pattern of HO-1 expression, that was enhanced by physical exercise in skeletal muscles of HO-1 transgenic mice, while downregulated in mdx counterparts.

At the tissue level, physical exercise-induced necrosis of selective skeletal muscle and associated increase in circulating levels of muscle damage markers was blunted in the animals that expressed *HMOX1*. SC-targeted *HMOX1* expression modulated muscle phenotype in transgenic mice as shown by 1) a decrease of serum level of muscle damage markers upon physical exercise, 2) decreased necrotic events in diaphragm, 3) in *TA* of trained mice (vs. mdx): decreased necrotic events, while increased (over mdx control) level of anti-inflammatory IL-10. In addition, anti-inflammatory effect of TX was detected in *TA* in untreated mdx mice as it decreased the necrotic events, although this was not seen in HO-1 transgenic mice.

We previously showed that HO-1 expression is induced in wild type mice in response to exercise or upon skeletal muscle injury as detected mainly in the infiltrating leukocytes [13, 14]. In mdx mice, enhanced level of HO-1 was detected in inflammatory cells and myofibers of limb skeletal muscles and diaphragm [15]. The genetic ablation of *Hmox1* (*Hmox1*^−/−^*mdx*) or pharmacological inhibition of HO activity aggravated muscle damage and inflammation in dystrophic mice and impaired exercise capacity [15]. Here, we confirmed HO-1 expression (detected by antibody recognizing murine and human antigen) in WBCs in both mdx;*HMOX1*^Pax7Ind^ and mdx mice, that was potentiated by physical exercise (significantly in mdx mice). Strikingly, nearly 90% of CD45^−^CD31^−^Sca-1^+^ cells, of both genotypes, expressed HO-1. The effect of physical exercise on the percentage of HO-1 positive cells in transgenic mice resembled the pattern outlined in HO-1^+^ SCs. It could be associated with the presence of side population (SP) SCs (syndecan-4^+^/ABCG2^+^/Sca-1^+^, Pax7^+^) among Sca-1^+^ population, that via Pax7 expression [20] would allow the induction of *HMOX1* expression.

Muscle biopsies from DMD patients [26] as well as myofibers [10] and hind limb skeletal muscles [27] isolated from mdx mice show elevated SC numbers relative to age-matched healthy controls. Such data contradicts the previously accepted theory of the SC exhaustion in the course of DMD, related to repetitive cycles of muscle degeneration and regeneration [11]. Here we show that TX-inducible increase of low basal level of HO-1 in SCs decreased SC number. It suggests the involvement of HO-1 in SC maintenance in DMD but requires mechanistical investigation.

As shown by us recently, although the global *Hmox1* knockout aggravated DMD pathology, it did not affect SC phenotype in mdx mice [15]. The lack of HO-1 did not significantly change neither the activation nor proliferation or differentiation of dystrophic SCs [15]. It could be associated with already low level of *Hmox1* expression detected in dystrophic SCs (in contrast to muscle fibers) [15]. We report here, that HO-1 transgenic mice show enhanced proliferation of SCs in response to physical exercise. It is in agreement with our previous data showing the increased survival and proliferation of HO-1 overexpressing C2C12 myoblasts upon intramuscular transplantation to NOD/SCID mice [17]. Accordingly, muscle-derived cells enriched in SCs obtained from *Hmox1*^−/−^ mice were more sensitive to H_2_O_2_ and showed decreased proliferation [17]. In contrast, cardiotoxin-induced muscle injury increased the rate of proliferation of SCs derived from skeletal muscles of *Hmox1*^*−/−*^ mice over *Hmox1*^+*/*+^ counterparts [14]. Thus, the HO-1 involvement in SC function may be dependent on the condition of the muscle and the injury model.

Studies of other groups have provided evidence that TX may act as a regulator of calcium homeostasis [28] and antioxidant, being efficient inhibitor of lipid peroxidation and intramembraneous scavenger of peroxyl radicals [29]. Data from murine models have revealed a role for TX in counteracting DMD pathology, attenuating muscle fibrosis and improving the muscle structure [30]. Our data show the anti-necrotic effect of TX mainly in *TA* of untreated mdx mice, but not in mdx;*HMOX1*^Pax7Ind^ mice. Such findings require more in-depth analysis. Nonetheless, the recent findings from the TX clinical trial show that although TX was safe and well tolerated, no difference between groups was concluded for the primary efficacy endpoint [31]. Due to insufficient clinical evidence, TX is currently not recommended to use in daily clinical practice [31].

## Conclusions

Here we showed that targeted expression of *HMOX1* plays protective role in DMD and alleviates dystrophic muscle pathology. HO-1 induction in SCs resulted in a drop of SC pool, the effect not visible in mdx controls, without affecting SC activation, that was increased by TX in HO-1 independent manner. HO-1 decreased the percentage of SCs in mdx;*HMOX1*^Pax7Ind^ mice to the comparable extent as the physical training. Upon physical exercise, the proliferation of SCs and activated CD34^−^ SC subpopulation was impaired in mdx mice*,* an effect that was reversed in mdx;*HMOX1*^Pax7Ind^ mice, however, both in vehicle- and TX-treated animals. This suggests the enhanced leakage of the Cre-LoxP system in exercise conditions resulting in enhanced *HMOX1* expression.

At the tissue level, targeted induction of HO-1 in SCs of mdx;*HMOX1*^Pax7Ind^ mice attenuated dystrophic muscle pathology. HO-1 transgenic mice showed lower serum level of exercise-induced muscle damage markers, blunted necrotic events of selective skeletal muscles and showed anti-inflammatory cytokine profile (vs. mdx).

### Supplementary Information

Supplementary file 1: Figure 1. (A) Body weight and (B) hind limb skeletal muscles weight of mdx and mdx;*HMOX1*^Pax7Ind^ mice (*n* = 5-9). Skeletal muscles from one limb (*caput gastrocnemius, soleus, tibialis anterior, EBL, quadriceps, adductor*) were collected 2 weeks after TX administration. Data is presented as mean^+/-^ SEM. ###* p* < 0.001 - Two-way ANOVA variation.

Supplementary file 2: Figure 2. (A) Schematic representation of SC gating strategy. (B) Quantitative analysis of the percentage of HO-1 positive cells among nucleated cells. Flow cytometry (*n* = 5-8). Data is presented as mean^+/-^ SEM.

Supplementary file 3: Figure 3. Quantitative analysis of the percentage of (A) CD45-CD31-, (B) CD45+CD31-b (WBC) , (C) CD45-CD31+ (EC), (D) CD45-CD31-Sca-1- and (E) CD45-CD31-Sca-1+ in hind limb skeletal muscles of mdx and mdx;*HMOX1*^Pax7Ind^ mice. Flow cytometry (*n* = 5-8). Data is presented as mean^+/-^ SEM. * *p* < 0.05 - vs. untr by two-way ANOVA with Tukey’s post hoc test. $ - *p* < 0.001 by unpaired two-tailed Student’s *t *test.

## Data Availability

The datasets used and analysed during the current study are available from the corresponding author on reasonable request.

## Abbreviations

*DIA*: *Diaphragmae*
*GAS*: *Caput gastrocnemius*
hiPSCs: Human-induced pluripotent stem cells
HO-1 (*HMOX1*, *Hmox1*): Heme oxygenase-1
*Il10*: Interleukin 10
SCs: Satellite cells
*TA*: *Tibialis anterior*
TX: Tamoxifen
