# Supplementary figures and images for: Targeted expression of heme oxygenase-1 in satellite cells improves skeletal muscle pathology in dystrophic mice
Source: Skelet Muscle. 2024 Jun 12;14:13. doi: 10.1186/s13395-024-00346-2 (PMC11167827; doi:10.1186/s13395-024-00346-2)

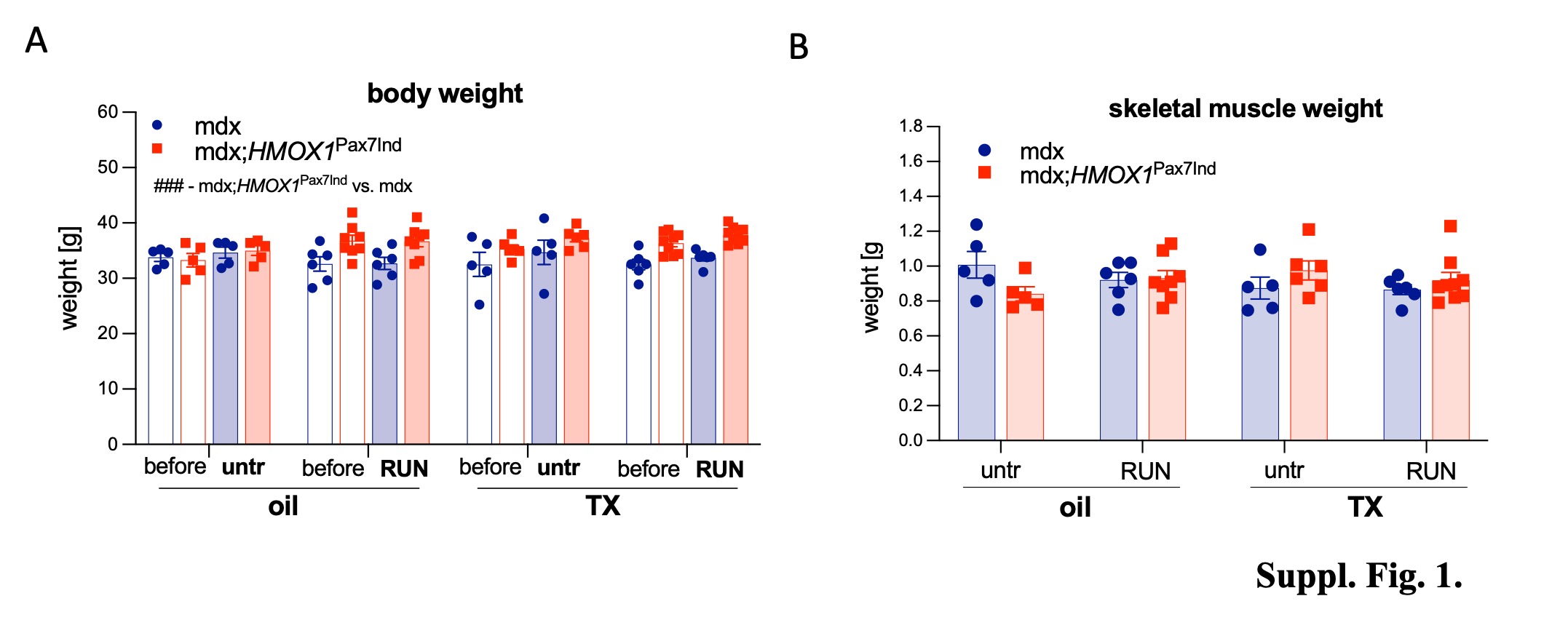

Supplement: Supplementary file 1 — Figure 1. (A) Body weight and (B) hind limb skeletal muscles weight of mdx and mdx;HMOX1Pax7Ind mice (n = 5-9). Skeletal muscles from one limb (caput gastrocnemius, soleus, tibialis anterior, EBL, quadriceps, adductor) were collected 2 weeks after TX administration. Data is presented as mean+/- SEM. ### p < 0.001 - Two-way ANOVA variation. [file 13395_2024_346_MOESM1_ESM.jpg]

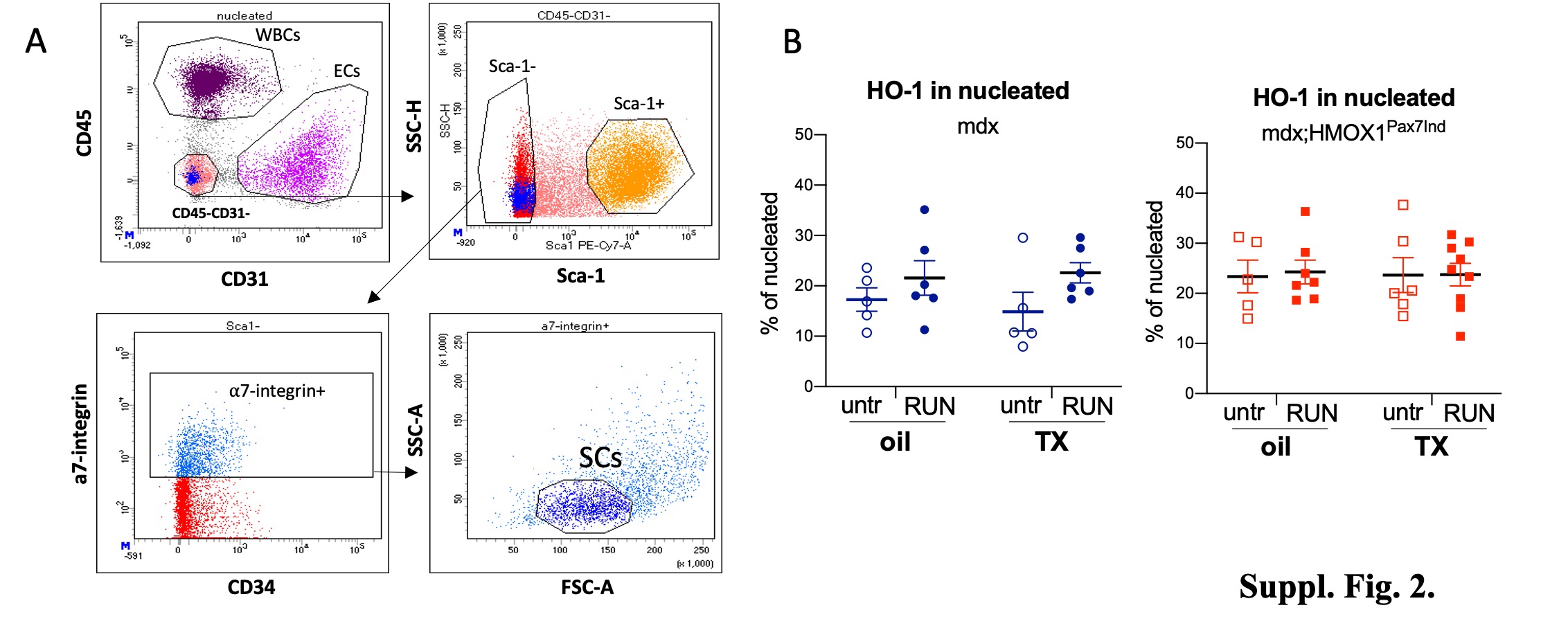

Supplement: Supplementary file 2 — Figure 2. (A) Schematic representation of SC gating strategy. (B) Quantitative analysis of the percentage of HO-1 positive cells among nucleated cells. Flow cytometry (n = 5-8). Data is presented as mean+/- SEM. [file 13395_2024_346_MOESM2_ESM.jpg]

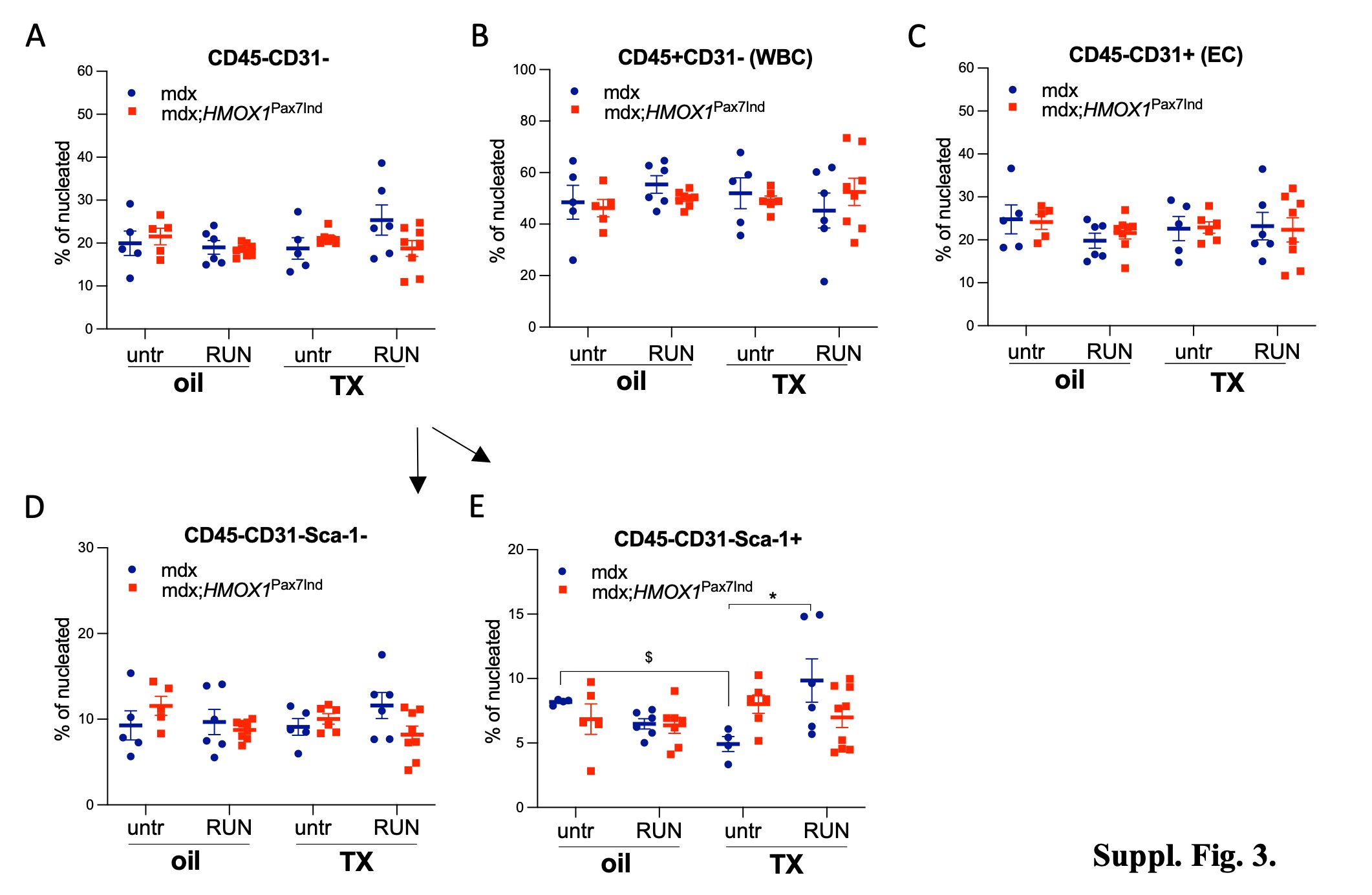

Supplement: Supplementary file 3 — Figure 3. Quantitative analysis of the percentage of (A) CD45-CD31-, (B) CD45+CD31-b (WBC) , (C) CD45-CD31+ (EC), (D) CD45-CD31-Sca-1- and (E) CD45-CD31-Sca-1+ in hind limb skeletal muscles of mdx and mdx;HMOX1Pax7Ind mice. Flow cytometry (n = 5-8). Data is presented as mean+/- SEM. * p < 0.05 - vs. untr by two-way ANOVA with Tukey’s post hoc test. $ - p < 0.001 by unpaired two-tailed Student’s t test. [file 13395_2024_346_MOESM3_ESM.jpg]
